# Immunogenic landscape and risk score prediction based on unfolded protein response (UPR)-related molecular subtypes in hepatocellular carcinoma

**DOI:** 10.3389/fimmu.2023.1202324

**Published:** 2023-06-30

**Authors:** Hanyao Guo, Sidi Zhang, Bo Zhang, Yanan Shang, Xiaoyu Liu, Meixia Wang, Hongyu Wang, Yumei Fan, Ke Tan

**Affiliations:** Ministry of Education Key Laboratory of Molecular and Cellular Biology; Hebei Research Center of the Basic Discipline of Cell Biology, Hebei Province Key Laboratory of Animal Physiology, Biochemistry and Molecular Biology, College of Life Sciences, Hebei Normal University, Shijiazhuang, Hebei, China

**Keywords:** unfolded protein response (UPR), hepatocellular carcinoma, molecular subtypes, immune microenvironment, drug sensitivity

## Abstract

**Background:**

Hepatocellular carcinoma (HCC) is the most common type of cancer and causes a significant number of cancer-related deaths worldwide. The molecular mechanisms underlying the development of HCC are complex, and the heterogeneity of HCC has led to a lack of effective prognostic indicators and drug targets for clinical treatment of HCC. Previous studies have indicated that the unfolded protein response (UPR), a fundamental pathway for maintaining endoplasmic reticulum homeostasis, is involved in the formation of malignant characteristics such as tumor cell invasiveness and treatment resistance. The aims of our study are to identify new prognostic indicators and provide drug treatment targets for HCC in clinical treatment based on UPR-related genes (URGs).

**Methods:**

Gene expression profiles and clinical information were downloaded from the TCGA, ICGC and GEO databases. Consensus cluster analysis was performed to classify the molecular subtypes of URGs in HCC patients. Univariate Cox regression and machine learning LASSO algorithm were used to establish a risk prognosis model. Kaplan–Meier and ROC analyses were used to evaluate the clinical prognosis of URGs. TIMER and XCell algorithms were applied to analyze the relationships between URGs and immune cell infiltration. Real time-PCR was performed to analyze the effect of sorafenib on the expression levels of four URGs.

**Results:**

Most URGs were upregulated in HCC samples. According to the expression pattern of URGs, HCC patients were divided into two independent clusters. Cluster 1 had a higher expression level, worse prognosis, and higher expression of immunosuppressive factors than cluster 2. Patients in cluster 1 were more prone to immune escape during immunotherapy, and were more sensitive to chemotherapeutic drugs. Four key UPR genes (ATF4, GOSR2, PDIA6 and SRPRB) were established in the prognostic model and HCC patients with high risk score had a worse clinical prognosis. Additionally, patients with high expression of four URGs are more sensitive to sorafenib. Moreover, ATF4 was upregulated, while GOSR2, PDIA6 and SRPRB were downregulated in sorafenib-treated HCC cells.

**Conclusion:**

The UPR-related prognostic signature containing four URGs exhibits high potential application value and performs well in the evaluation of effects of chemotherapy/immunotherapy and clinical prognosis.

## Introduction

Liver cancer has become the main cause of cancer-associated deaths worldwide. It is predicted that 1.3 million people will die of liver cancer by 2040, an increase of 56.4% over 2020 (1). As the main type, primary liver cancer can be divided into hepatocellular carcinoma (HCC), intrahepatic cholangiocarcinoma (ICC), and combined hepatocellular carcinoma and cholangiocarcinoma (cHCC-CC). At present, the therapeutic strategies for HCC mainly include surgery, chemotherapy, radiotherapy and immunotherapy (2–5). Unfortunately, the survival rate of HCC patients is still not satisfactory (6). With the development of bioinformatics, an increasing number of prognostic biomarkers of HCC have been discovered, and these biomarkers have been applied to the clinical prognosis of HCC (7).

Cancer cells are challenged by various environmental and oncogenic stresses during oncogenesis and metastasis, and are required to meet the increased needs for protein generation for rapid growth and proliferation. To overcome these challenges, cancer cells exploit a distinct series of adaptive molecular mechanisms, including heat shock response, mitochondrial unfolded protein response and unfolded protein response (UPR, also referred as endoplasmic reticulum stress) (8–11). The endoplasmic reticulum (ER) is a multifunctional organelle in cells that performs multiple functions, including protein processing, maturation and transportation. When unfolded or misfolded proteins are over accumulated in the ER, UPR is activated to slow the synthesis of overall proteins, and enhances the expression of chaperones to increase the folding capacity (12). In mammals, UPR signaling pathways are orchestrated by three ER-localized proteins: protein kinase R (PKR)-like ER kinase (PERK), inositol requiring enzyme 1 (IRE1) and activating transcription factor 6 (ATF6) (13, 14). Under normal conditions, these proteins are maintained in an inactive state. When unfolded or misfolded proteins accumulate in the ER, these three proteins are activated to modulate the downstream signaling pathways and effectors of UPR to alleviate the ER workload.

An increasing number of studies have indicated that UPR is significantly involved in many physiological processes, such as promoting cell survival, regulating angiogenesis and modulating the immune response during cancer progression (15). Therefore, UPR is closely associated with the progression of multiple human diseases, including heart diseases, neurodegenerative diseases and cancer (15). Importantly, UPR activation also promotes the development of drug resistance in cancer cells, making it an important target for cancer therapy. Three UPR regulators (PERK, IRE1 and ATF6) not only govern the switch between pro-survival and pro-death signals but also closely correlate with several hallmarks of cancer. UPR caused by ER homeostasis imbalance is one of the factors contributing to tumorigenesis by activating adaptive and survival pathways, but too long or too serious UPR would lead to apoptosis of tumor cells (16). Of note, cancer immunotherapy targeting UPR is gradually emerging (17). UPR plays an important role in the development, activation and homeostasis of T cells, thus affecting their function in immunotherapy (18, 19). UPR has been applied to the clinical prognosis of acute myeloid leukemia and has achieved good results (20). UPR also performs well as a prognostic indicator for bladder cancer and osteosarcoma (21, 22). Thus, UPR can be used as potential therapeutic target for different tumors. Deep understanding of UPR signaling pathways in oncology and clarifying the potential of UPR-targeting drugs could improve cancer treatment.

In this study, we used UPR-related genes (URGs) to analyze the precise roles of UPR pathway in the progression of HCC, as well as its immune infiltration characteristics and correlation with drug sensitivity. According to the expression of URGs, we used consensus clustering to divide the patients into two subgroups. We analyzed the mutation maps, signaling pathways, and differences in immune infiltration characteristics in two distinct clusters. We built a risk prognostic model and predicted drug sensitivity. The high-risk group corresponded to a lower survival rate and higher chemotherapeutic responses, which suggested that URGs had potential application value and guided clinical therapies in the clinical treatment of HCC patients. Meanwhile, we identified four core URGs with high prognostic value in HCC and validated that sorafenib affected the expression of these four URGs. The flow chart of this study is shown (**Figure S1**).

## Materials and methods

### Data collection

The mRNA expression data and clinical profiles were provided by the TCGA (The Cancer Genome Atlas) database (https://portal.gdc.cancer.gov), ICGC (International Cancer Gene Consortium) database (https://dcc.icgc.org/releases/current/Projects) and GEO (Gene Expression Omnibus) database (https://www.ncbi.nlm.nih.gov/geo/). In the TCGA database, we collected 371 HCC samples and 50 normal samples. In the ICGC database, we collected 240 HCC samples and 202 normal samples. GSE14520 and GSE36376 datasets were used to analyze the expression changes of four key URGs in normal and HCC tissues. The TNMplot database (https://tnmplot.com/analysis/) was used to compare the expression of UPR-related signature in HCC tissues and normal tissues.

### Establishment of the UPR-related gene set

In the TCGA dataset, |Log_2_(fold change)| > 0.58 and *P <* 0.05 were set as a threshold to screen the differentially expressed genes (DEGs) between HCC and normal samples. Then, we obtained genes associated with UPR in the MSigDB (http://software.broadinstitute.org/gsea/msigdb/index.jsp) and UALCAN (http://ualcan.path.uab.edu) databases, and intersected them with DEGs to finally obtain 31 URGs for establishing a UPR-related gene set. The R language packages “ggplot2”, “ggunchained” and “pheatmap” were used. Signaling pathway enrichment analysis was completed by the Metascape database (https://metascape.org/gp/index.html#/main/step1).

### Consensus clustering analysis of URGs

The R language package “ConsensusClusterPlus” was used to investigate the underlying molecular clusters based on the expression of 31 URGs. Principal component analysis (PCA) was used to evaluate the expression patterns of URGs. The R package “pheatmap” was used to draw a clustering heatmap. The R packages “survival” and “survminer” were used for Kaplan–Meier (KM) analysis.

### Univariate Cox and LASSO Cox regional analyses were used to establish risk scoring models

Univariate Cox analysis was utilized to screen prognosis-related UPRs based on the TCGA and ICGC databases. Least absolute shrinkage and selection operator (LASSO) regional analysis was further carried out to build a risk scoring model with the R language package “glmnet”. In the risk scoring model, risk score = sum of coefficients × prognostic URG expression. The HCC samples were separated into high-risk and low-risk groups by means of the median risk score as the cutoff point. KM analysis was performed to assess the differences in prognosis using the log-rank test. Receiver operating characteristic (ROC) curves were generated using the R package “timeROC” to evaluate the accuracy of the risk scoring model.

### Construction of a nomogram

The R language package “forestplot” was used to construct the forest plot and display variables such as *P* value, HR and 95% CI in regression analysis. The “rms” package was used to make nomogram diagrams based on URG expression, age tumor (T), node (N), metastasis (M) and grade. The predictive and discriminative ability of the nomogram for 1-, 3-, and 5-year OS was assessed using ROC and concordance index (C-index) using the R package “survival”. Calibration curves were used to evaluate the difference between predicted results and actual survival rate of patients.

### Genomic mutation profiles of two clusters

The R language package “maftools” was applied to visualize the mutation maps of two clusters and generate the waterfall plots.

### Signaling pathway enrichment analysis of DEGs between two clusters

The R language package “limma” was used to select DEGs between cluster 1 and cluster 2. |Log_2_(fold change)| > 0.58 and *P <*0.05 were used as thresholds. The “ClusterProfiler” package was used for Gene Ontology (GO) and Kyoto Encyclopedia of Genes and Genomes (KEGG) pathway analysis of DEGs between cluster 1 and cluster 2.

### Abundant analysis of immune cell infiltration

The R language package “immunedecov” was utilized to calculate the infiltrated abundance of immune cells in two clusters according to the XCell and TIMER algorithms. The “pheatmap” package was used to display the abundance of immune cells in each HCC sample.

### Analysis of immunotherapy response

We analyzed the difference in the expression of common immune checkpoint genes between cluster 1 and cluster 2, and used the “ggplot2” and “ggpubr” packages for visualization. The tumor immune dysfunction and exclusion (TIDE) algorithm (https://tide.dfci.harvard.edu) was used to evaluate the difference in immunotherapy response between two clusters.

### Single-cell RNA-sequencing and immunohistochemical analysis

Immunohistochemical staining (IHC) results of normal liver tissues and HCC tissues were obtained from the Human Protein Atlas (HPA) database (https://www.proteinatlas.org/). To verify the expression of core URG expression in different cells in HCC tissues, single-cell RNA-sequencing results were obtained from the Human Liver Browser (https://itzkovitzwebapps.weizmann.ac.il/webapps/home/session.html?app=HumanLiverBrowser).

### Prediction of therapeutic effect of chemotherapeutic drugs

The Genomics of Drug Sensitivity in Cancer (GDSC) database (https://www.cancerrxgene.org/) and R language package “pRRophetic” were used to evaluate the sensitivity of two clusters to chemotherapeutic drugs, and the half maximum inhibitory concentration (IC50) was calculated by ridge regression. The correlations between IC50 and the risk score and expression of core URGs were evaluated.

### Real-time polymerase chain reaction (RT-PCR)

Human hepatocellular carcinoma cell lines HepG2 and Huh7 were cultured in Dulbecco’s modified Eagle’s medium (DMEM) supplemented with 10% fetal bovine serum (FBS) and 1% penicillin/streptomycin in a humid atmosphere (37°C, 5% CO_2_) as described previously (11, 23, 24). HepG2 and Huh7 cells were treated with different concentrations of sorafenib for 24 h. Total RNA was extracted using the RNA-easy kit (Vazyme, Nanjing, China). Reverse transcription kit (Biosharp, Beijing, China) was used to reverse transcribe RNA into cDNA. RT-PCR was performed using SYBR qPCR Master Mix (Biosharp, Beijing, China) to examine the expression levels of four target genes according to the manufacturer’s instruction. The primer sequences are shown in **Table 1**. Relative mRNA level was calculated by 2^-ΔΔCT^ method normalized with internal reference gene S18.

**Table 1:** Primers used for RT-PCR.

Gene	Forward primers	Reverse primers
ATF4	CTCCAACATCCAATCTGTCCCG	TTCTCCAGCGACAAGGCTAAGG
GOSR2	CAGACCTTCCTCCAAAGTGTGC	ATGCTGGGCTTGTCCAACACAG
PDIA6	CCTCTTGGCAATGTCCTCGTTG	TCAGAAAGGCGAGTCTCCTGTG
SRPRB	GGACTTGATACAGAAACTCAGCC	GAGGCTTCAGTTCTTAGAGCGG
S18	GTTCCGACCATAAACGATGCC	TGGTGGTGCCCTTCCGTCAAT

### Statistical methods

All statistical analyses were completed by R software (version 4.2.2). The Wilcoxon test was used to compare the significant differences between two groups. The Kruskal–Wallis test was applied to estimate the differences among three groups. The log-rank test was used for KM analysis. Pearson correlation analysis was used to measure the correlation between the two groups. *P* < 0.05 was defined as a statistically significant difference.

## Results

### Collection of UPR-related genes

**Figure 1:**
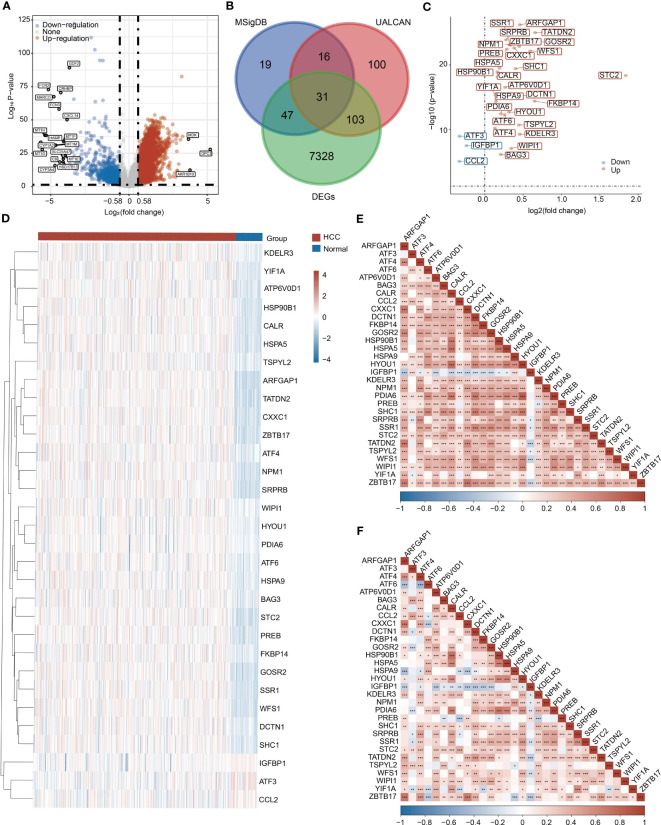
Collection of UPR-related genes (URGs). **(A)** Identification of DEGs between HCC samples and normal samples in the TCGA database. **(B)** Venn diagram showing the screening of URGs. **(C)** Differentially expressed 31 URGs in HCC and normal samples. **(D)** Heatmap showing the expression of 31 URGs in HCC and normal samples. **(E)** A correlation heatmap of 31 URGs based on the TCGA database. **(F)** A correlation heatmap of 31 URGs based on the ICGC database. *P < 0.05, **P < 0.01, ***P < 0.001.

To obtain differentially expressed UPR-related genes (URGs), we comprehensively considered the following three aspects. First, we collected mRNA expression data from 371 HCC samples and 50 normal samples in the TCGA database. A total of 7509 DEGs were selected according to |Log_2_(fold change)| > 0.58 and *P* < 0.05 (**Figure 1A**, **S2A**). Second, we retrieved 113 and 250 URGs from the MSigDB and UALCAN databases, respectively. Finally, we selected the intersection of three gene sets as differentially expressed URGs, and a total of 31 URGs were collected (**Figure 1B**).

### Differential expression of URGs in HCC

We investigated the expression of 31 URGs in HCC and normal samples using the TCGA database. Among 31 URGs, ARFGAP1, ATF4, ATF6, ATP6V0D1, BAG3, CALR, CXXC1, DCTN1, FKBP14, GOSR2, HSP90B1, HSPA5, HSPA9, HYOU1, KDELR3, NPM1, PDIA6, PREB, SHC1, SRPRB, SSR1, STC2, TATDN2, TSPYL2, WFS1 and WIPI1 were significantly upregulated in HCC samples (**Figures 1C, D** and **Figure S2B**). The expression of ATF3, CCL2 and IGFBP1 was obviously downregulated in HCC compared with normal samples (**Figures 1C, D** and **Figure S2B**).

To further validate the reliability of the above conclusion, we performed similar analysis using the ICGC database. ARFGAP1, ATF4, ATF6, ATP6V0D1, HYOU1, KDELR3, NPM1, PDIA6, PREB, SHC1, SRPRB, SSR1, STC2, TATDN2, WFS1, WIPI1, YIF1A and ZBTB17 expression was higher in HCC samples, while ATF3, CCL2, IGFBP1 and TSPYL2 expression was lower in HCC samples than normal samples (**Figures S3A**, **S3B**). Collectively, most URGs were upregulated in HCC samples.

### Interaction and correlation analysis of URGs

To further understand the relationships, signaling pathways and molecular mechanisms of URGs, we used Metascape tool to perform signaling pathway enrichment analysis based on 31 URGs. Consistent with our speculation, these URGs were mainly enriched in a series of pathways related to ER stress, such as unfolded protein response (UPR) and cellular response to unfolded protein (**Figure S4**). URGs were also associated with immunity-related pathway, such as IL-18 signaling pathway (**Figure S4**).

The correlations among URGs were further calculated based on the TCGA and ICGC databases, and we observed a large number of significant correlations between URGs, of which positive correlations accounted for the majority (**Figures 1E, F**).

### Consensus clustering analysis of URGs in HCC samples

**Figure 2:**
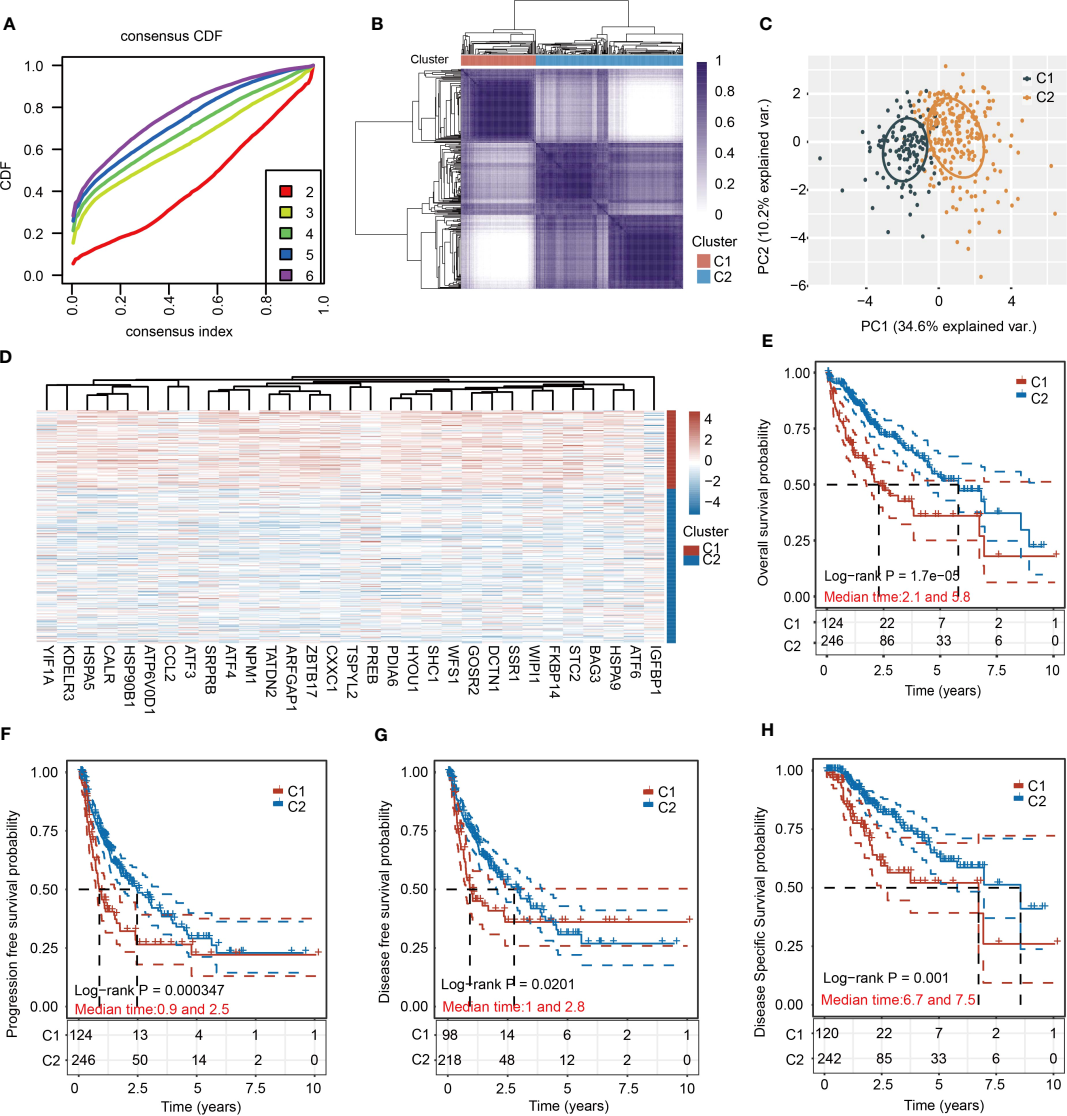
Cluster analysis of HCC samples based on the 31 URGs expression. **(A)** Cumulative distribution function curves. **(B)** Consensus clustering matrix when k=2. **(C)** PCA of HCC samples according to the expression of 31 URGs. **(D)** Heatmap showing the expression of 31 URGs in C1 and C2. **(E–H)** KM analysis of OS, PFS, DFS and DSS in C1 and C2.

To apply URGs to personalized treatment of HCC patients, we classified the molecular subtypes of patients based on the expression levels of 31 URGs. We increased the number of clusters (k) from 2 to 6, and found that the well clustering effect was obtained when k = 2 (**Figure 2A**). Therefore, HCC samples were divided into two clusters (**Figure 2B**). The PCA analysis shows that the two clusters are well separated (**Figure 2C**). The expression of URGs in cluster 1 (C1) was generally higher than that in cluster 2 (C2) (**Figure 2D**). To determine the prognostic value of molecular classification, we then used KM analysis to analyze the overall survival (OS), progression-free survival (PFS), disease-free survival (DFS) and disease-specific survival (DSS) of HCC patients. Patients in C1 had worse prognosis than those in C2 (**Figures 2E–H**). Furthermore, HCC patients in C1 and C2 exhibited significant differences in terms of T stage, TNM stage and grade (**Table 2**).

**Table 2:** Relationships between various clinicopathological parameters and two clusters in HCC.

	Characteristic	C1	C2	*P* value
**Status**	Alive	70	171	
	Dead	55	75	0.014
**Age**	Mean (SD)	58.1 (13.2)	60.1(13.2)	
	Median [MIN, MAX]	59 (18,85)	62 (16,90)	0.183
**Sex**	FEMALE	41	80	
	MALE	84	166	1
**Race**	AMERICAN INDIAN	1	1	
	ASIAN	56	102	
	BLACK	6	11	
	WHITE	62	122	0.954
**pT-stage**	T1	44	137	
	T2	40	52	
	T2a	1		
	T2b	1		
	T3	19	26	
	T3a	11	18	
	T3b	2	4	
	T4	7	6	
	TX		1	0.01
**pN-stage**	N0	82	170	
	N1	3	1	
	NX	39	75	0.2
**pM-stage**	M0	90	176	
	M1	1	3	
	MX	34	67	0.933
**pTNM-stage**	I	43	128	
	II	36	50	
	III	1	2	
	IIIA	27	38	
	IIIB	4	4	
	IIIC	6	3	
	IV	1	1	
	IVA		1	
	IVB		2	0.018
**Grade**	G1	11	44	
	G2	53	124	
	G3	54	68	
	G4	6	6	0.004

### Identification of DEGs and gene enrichment analyses between C1 and C2

**Figure 3:**
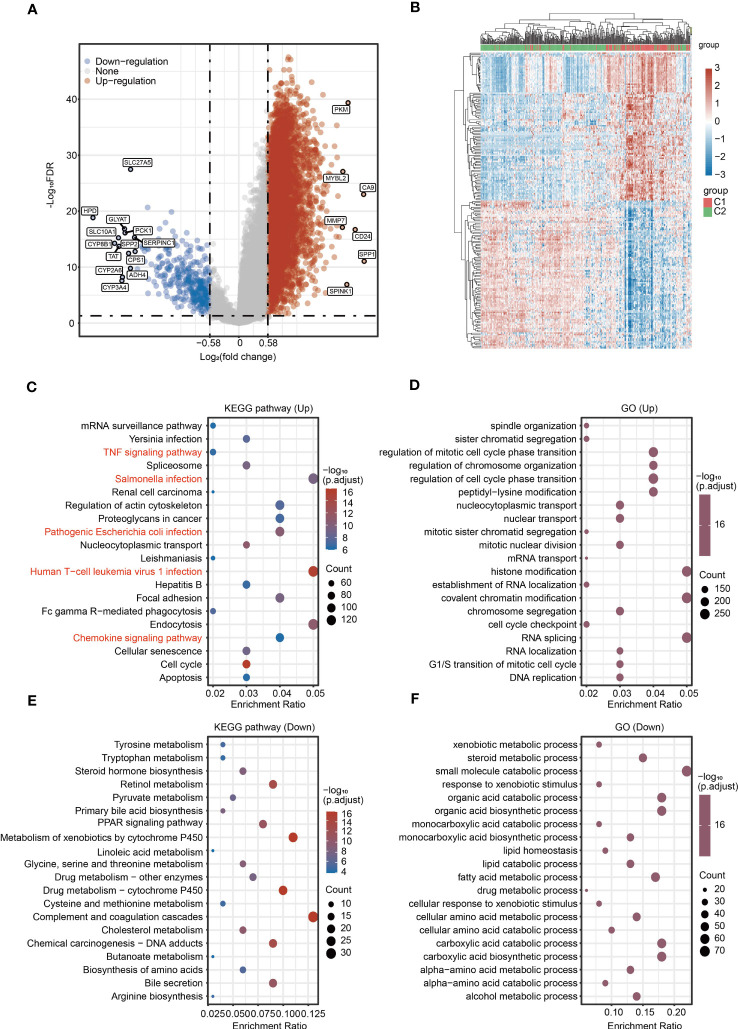
Identification of DEGs and signaling pathway enrichment analysis for DEGs. **(A)** Volcano plot showing the DEGs between C1 and C2. **(B)** Heatmap showing the DEGs between C1 and C2. **(C, D)** KEGG and GO enrichment analyses for the upregulated genes. **(E, F)** KEGG and GO enrichment analyses for the downregulated genes.

To provide a more comprehensive comparison between two molecular subtypes, we performed differential expression analysis, GO and KEGG analyses between C1 and C2. Upregulated genes and downregulated genes were identified based on |Log2(fold change)| > 0.58 and *P* < 0.05 (**Figures 3A, B**). The top five KEGG pathways for upregulated genes were *Salmonella* infection, human T−cell leukemia virus 1 infection, endocytosis, focal adhesion, and pathogenic *Escherichia coli* infection (**Figure 3C**). The top five GO pathways for upregulated genes were histone modification, covalent chromatin modification, RNA splicing, regulation of mitotic cell cycle phase transition, and regulation of cell cycle phase transition (**Figure 3D**). The top five KEGG pathways for downregulated genes were complement and coagulation cascades, metabolism of xenobiotics by cytochrome P450, drug metabolism-cytochrome P450, chemical carcinogenesis-DNA adducts, and bile secretion (**Figure 3E**). Additionally, the top five GO pathways for downregulated genes were small molecule catabolic process, organic acid catabolic process, organic acid biosynthetic process, carboxylic acid catabolic process, and carboxylic acid biosynthetic process (**Figure 3F**). Of note, several immunity-related pathways were also enriched in the KEGG results, including TNF signaling pathway, *Salmonella* infection, pathogenic *Escherichia coli* infection, human T-cell leukemia virus 1 infection, and chemokine signaling pathway (**Figure 3C**).

Ferroptosis is a new type of programmed cell death and N6-methyladenosine (m6A) methylation is a common modification in eukaryotic mRNA. Both ferroptosis and m^6^A methylation play key roles in tumorigenesis, metastasis and immune escape in HCC. We then analyzed the expression of ferroptosis-related genes and m^6^A-related genes between C1 and C2. Most ferroptosis-related genes and m^6^A-related genes were upregulated in C1 compared with C2 (**Figures S5A**, **S5B**).

### Immune cell infiltration analysis between C1 and C2

**Figure 4:**
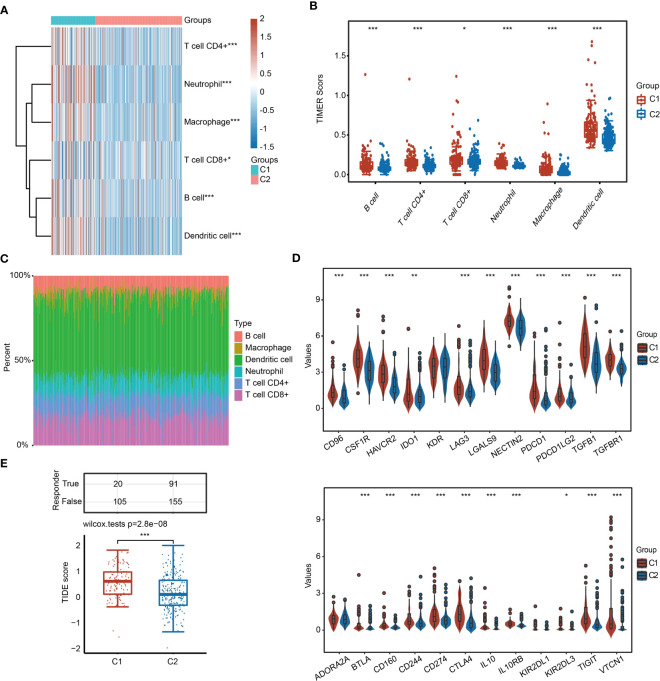
Infiltrated abundance of immune cells based on the TIMER algorithm in C1 and C2. **(A, B)** Comparison of infiltrated abundance of six types of immune cells in C1 and C2. **(C)** Bar chart showing the percentage of abundance of six immune cells in each HCC sample. **(D)** Violin diagram showing the difference in the expression of immunosuppressive genes in C1 and C2. **(E)** TIDE score of C1 and C2. *P < 0.05, **P < 0.01, ***P < 0.001.

Because identified 31 URGs and DEGs between C1 and C2 were involved in immunity-related pathways (**Figure 3C**), we further evaluated the differences in the immune microenvironment between two subgroups. The TIMER algorithm was used to assess the abundance of infiltrating immune cells in C1 and C2 subgroups. Except for CD8+ T cells, the abundance of five major immune cells in C1 was significantly higher than that in C2 (**Figures 4A, B**). Among the immune microenvironments of all HCC samples, dendritic cells (DCs) accounted for the largest proportion (**Figure 4C**). To further verify the accuracy of the conclusion, we performed similar analysis using the XCell algorithm. Among the 28 types of immune cells, 16 types exhibited significant differences in infiltrated abundance of immune cells between C1 and C2 (**Figures S6A–C**). Additionally, the expression of immunosuppressive factors, including CD96, CSF1R, HAVCR2, IDO1, LAG3, LGALS9, NECTIN2, PDCD1, PDCD1LG2, TGFB1, TGFBR1, BTLA, CD160, CD244, CD274, CTLA4, IL10, IL10RB, TIGIT and VTCN1, was significantly higher in C1 than that in C2 (**Figure 4D**). To further estimate the responses to immunotherapy, TIDE algorithm was used. HCC patients with lower TIDE score may respond better to immunotherapy. Notably, the TIDE score of C1 was significantly higher than C2, indicating that HCC patients in C2 were more sensitive to immunotherapy compared with C1 (**Figure 4E**).

### Genetic mutation of C1 and C2 in HCC

**Figure 5:**
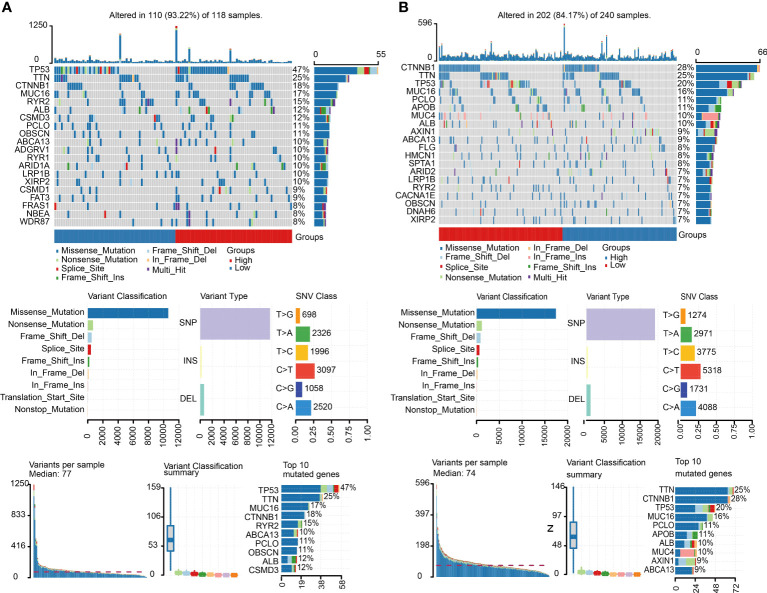
Mutation landscape of C1 and C2. **(A)** Summary of the variation landscape of C1, including variation type and classification and SNV classification. **(B)** Summary of the variation landscape of C2.

We obtained the mutation information of C1 and C2 from the TCGA database. The waterfall plot results demonstrated that the top five genes with the high frequency of alteration in C1 were TP53, TTN, CTNNBI, MUC16 and RYR2 (**Figure 5A**), and the top five genes with high frequency of mutation in C2 were CTNNB1, TTN, TP53, MUC16 and PCLO (**Figure 5B**). Missense mutation was the most frequent mutation type, in which single-nucleotide polymorphism (SNP) was the main part. SNV class showed that the most common variation was C > T in both subgroups.

### Prediction of drug sensitivity between C1 and C2 subgroups

**Figure 6:**
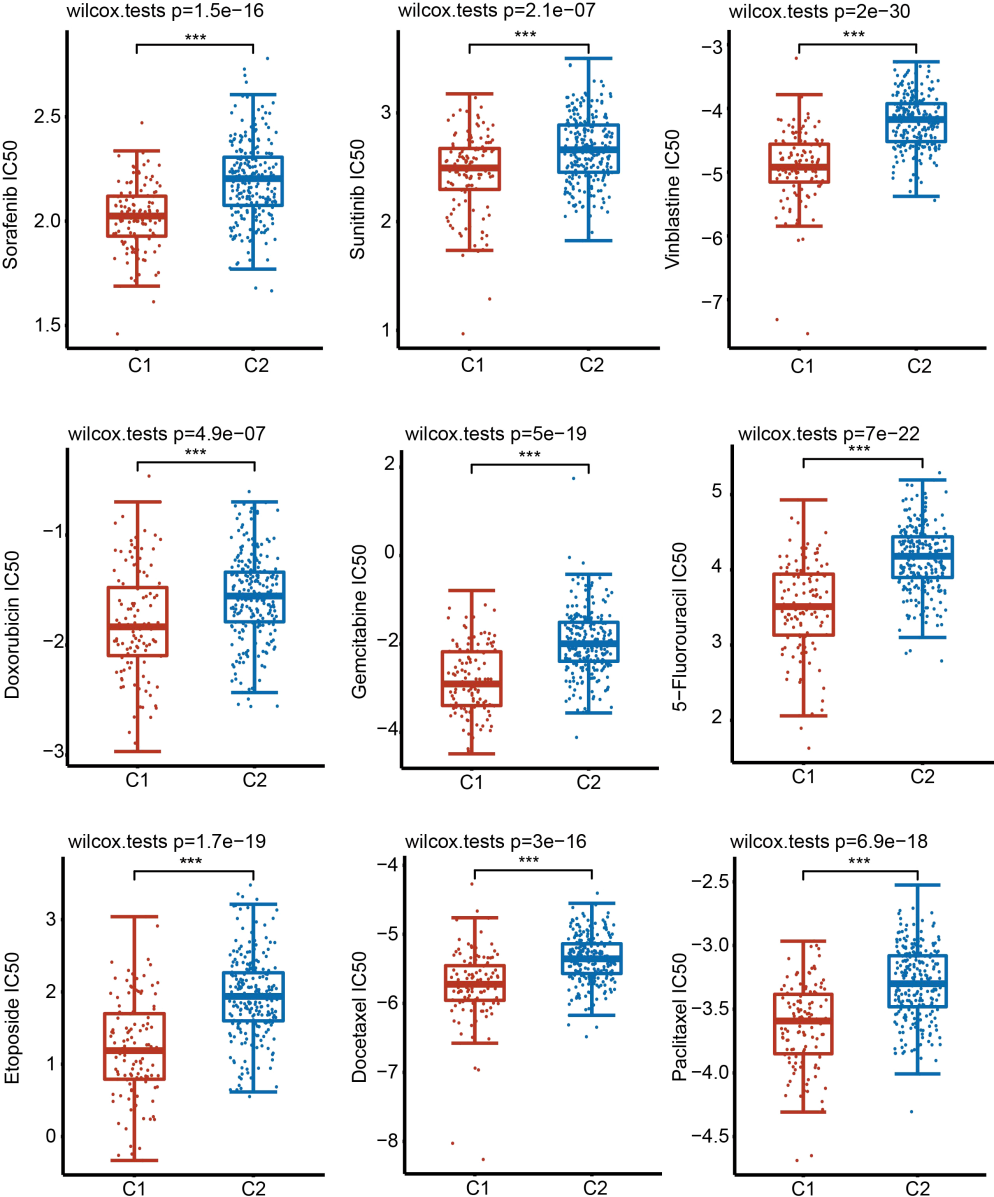
Sensitivity of 9 common chemotherapy drugs. Comparison of the sensitivity of 9 drugs in C1 and C2 by examining the IC50 values. ***P < 0.001.

Considering the clinical importance of chemotherapy, we then evaluated the sensitivity of 9 common chemotherapeutic drugs between two subgroups based on the GDSC database. The IC50 of 9 chemotherapeutic drugs, including sorafenib, sunitinib, vinblastine, doxorubicin, gemcitabine, 5-fluorouracil, etoposide, docetaxel and paclitaxel, in C1 was significantly lower than that in C2 (**Figure 6**). These results imply that although HCC patients in C1 have a lower survival rate than patients in C2, fortunately, these patients were more sensitive to chemotherapy.

### Construction of a URG-related prognostic signature in the TCGA database

**Figure 7:**
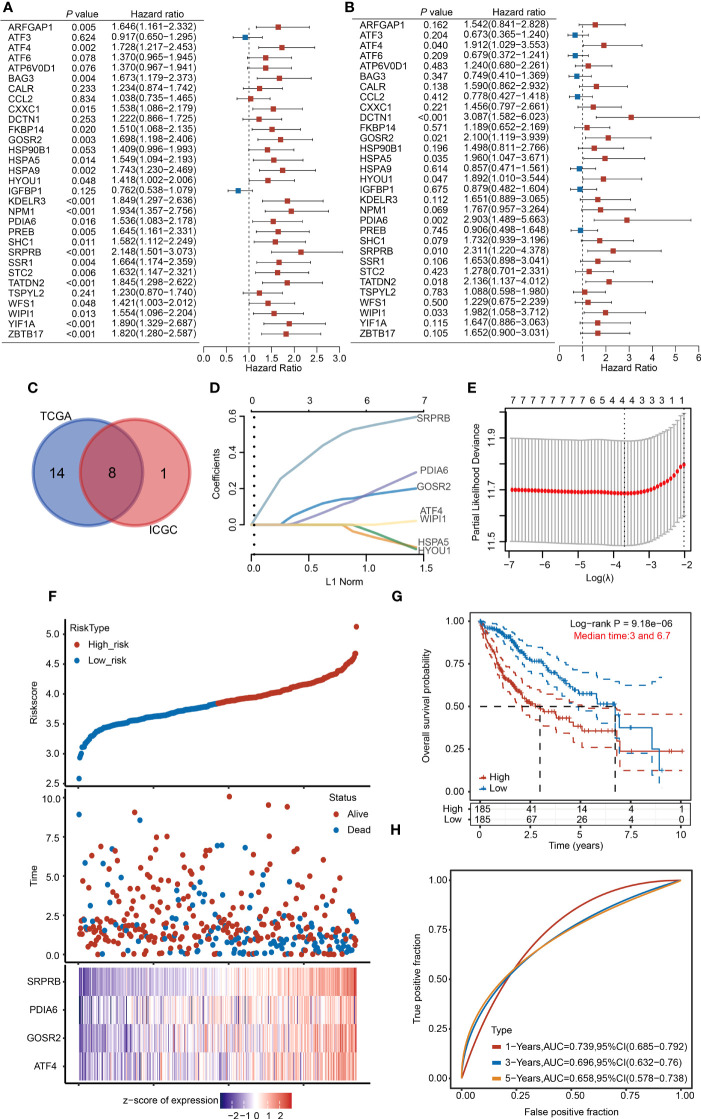
Establishment of the 4-URGs risk prognostic model in the TCGA database. **(A, B)** Forest plot showing the prognostic value of URGs using the univariate Cox regression analysis in the TCGA database and ICGC database. **(C)** Intersection of prognostic genes screened from TCGA and ICGC databases. **(D, E)** The LASSO algorithm was performed to establish the prognostic model and screen out four key URGs. **(F)** According to the risk scoring model established by four URGs, HCC patients were divided into high-risk and low-risk subgroups. **(G)** KM analysis of OS in high-risk group and low-risk subgroup. **(H)** AUC of time-dependent ROC curves was examined to test the reliability of the risk scoring model.

To further screen for URGs with prognostic value and better apply URGs to the clinical diagnosis of HCC patients, we performed a univariate Cox regression analysis and selected genes with prognostic potential (P < 0.05) (**Figures 7A, B**). 22 genes were selected from the TCGA dataset and 9 genes were selected from the ICGC dataset. Finally, 8 genes, including SRPRB, PDIA6, GOSR2, ATF4, WIPI1, HSPA5 and HYOU1, were selected from the intersection for subsequent analysis (**Figure 7C**).

We then used the optimum λ value to reduce the dimension of 8 genes by LASSO and established a prognostic risk model. According to the LASSO algorithm, we identified 4 core prognostic genes: ATF4, PDIA6, GOSR2 and SRPRB (**Figures 7D, E**). Based on the expression levels of 4 URGs, the risk score was calculated through the following formula: risk score = (0.0196×ATF4) + (0.1309×GOSR2) + (0.0889×PDIA6) + (0.4707×SRPRB). According to the median risk score, we divided HCC patients into high-risk group and low-risk group. The risk score, survival time and expression of four URGs in each patient were shown (**Figure 7F**). KM analysis results indicated that the survival rate of high-risk group was significantly lower than low-risk group (**Figure 7G**). We then used receiver operating characteristic (ROC) analysis to test the reliability and sensitivity of this prognostic model. The 1-year survival rate of areas under the ROC curve (AUC) was 0.739, the 3-year survival rate of AUC was 0.696, and the 5-year survival rate of AUC was 0.658 (**Figure 7H**). The above results indicate that the prognostic model we established is reliable.

To make our model more convincing, we further established a multivariate Cox regression analysis model using the four core genes using the ICGC database (**Figure S7A**). The survival rate of patients with high risk scores was worse than that of patients with low risk scores (**Figure S7B**). The AUC scores in the validation cohort were 0.815, 0.693 and 0.678 for 1-, 2-, and 3 years, respectively (**Figure S7C**).

To further verify the prediction accuracy of the 4-URG prognostic model, we carried out univariate and multivariate Cox regression analyses. Univariate Cox regression analysis indicated that ATF4, GOSR2, PDIA6, SRPRB, T stage, M stage and grade were significantly correlated with OS (**Figure S8A**). Multivariate Cox regression analysis suggested that SRPRB and T stage were markedly correlated with OS (**Figure S8B**). According to the results of multivariate Cox regression analysis, we used SRPRB and T stage to build a nomogram model to predict the OS of HCC patients at 1-, 3-, and 5 years (**Figure S8C**). Calibration plots showed agreement between actual and predicted survival rates (**Figure S8D**).

### Relationships between the risk model and clinicopathological characteristics

To assess the prognostic value of the 4-URG risk model in clinical practice, we analyzed the relationships between risk scores and various clinicopathological parameters, including age, sex, early grade (G1+G2) and advanced grade (G3+G4), early stage (T1+T2) and advanced stage (T3+T4), M0 and N0, TNM stage I+II and TNM stage III+IV. In the above clinicopathological parameters, the OS of high-risk group was worse than that of low-risk group (**Figure S9**). These results proved that 4-URGs have good potential application value in the clinical prognosis of HCC.

### Expression of 4 URGs

**Figure 8:**
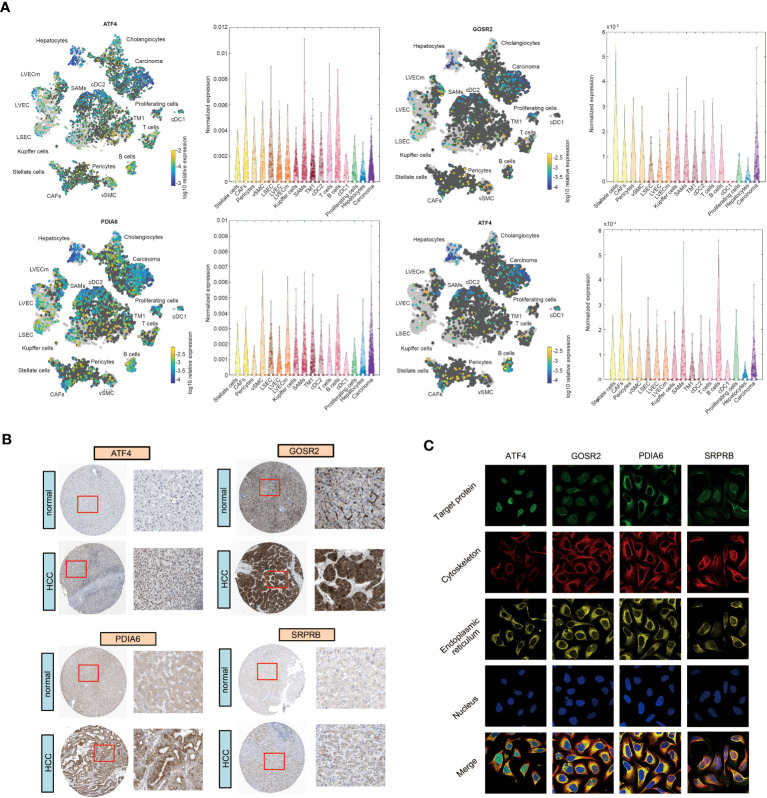
The expression and subcellular localization of four URGs. **(A)** Single-cell RNA sequencing results of four URGs. **(B)** IHC of four URGs in normal and HCC tissues. **(C)** Subcellular localization of four URGs.

To further verify the expression pattern of four URGs in liver cells, we collected single-cell RNA-sequencing data from the Human Life Browser database. Four URGs were expressed not only in HCC cells but also in immune cells (**Figure 8A**). The expression of 4-URG signature was not only upregulated in HCC tissues, but also much higher in metastatic tissues (**Figure S10A**, **S10B**). We also analyzed the expression differences of four URGs in the GSE36376 and GSE14520 datasets. As expected, the expression of four URGs was markedly elevated in HCC samples (**Figure S10C**, **S10D**). The HPA database was then used to compare the protein expression of four URGs. IHC results showed that the protein levels of ATF4, GOSR2, PDIA6 and SRPRB in HCC tissues were obviously higher than normal tissues (**Figure 8B**). We also collected immunofluorescence results in the HPA database to analyze subcellular localization. The results demonstrated that ATF4 was mainly located in the nucleus, GOSR2 was located in the Golgi apparatus, PDIA6 was located in the ER, and SRPRB was mainly located in the ER membrane (**Figure 8C**).

### Effect of sorafenib treatment on the expression of 4 URGs

**Figure 9:**
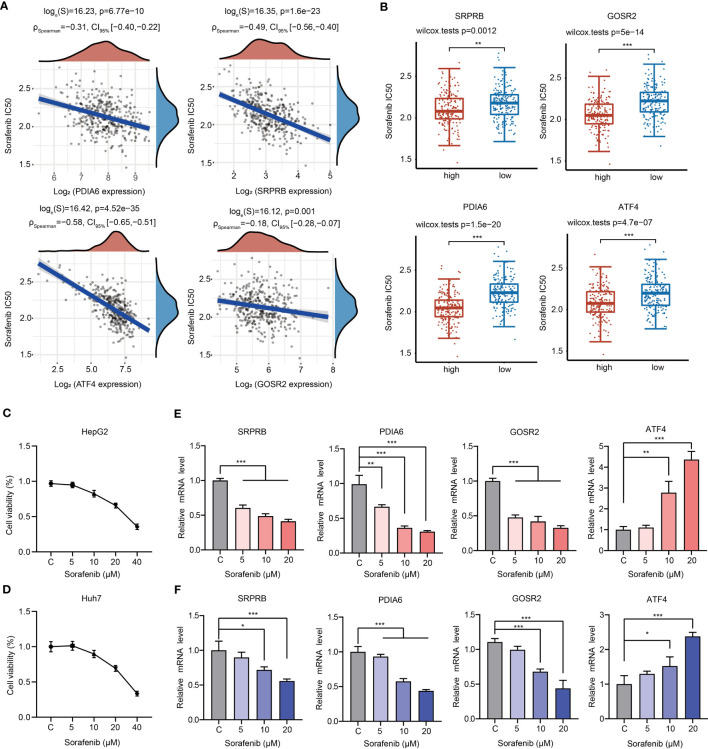
Relationships between 4 URGs and sorafenib. **(A)** Relationship between the expression of four URGs and the IC50 value of sorafenib. **(B)** Comparison of sorafenib IC50 values between URGs high-expression group and URGs low-expression group. **(C, D)** The cell viability of HepG2 and Huh7 cells under sorafenib treatment was examined, respectively. **(E, F)** The effect of sorafenib on the expression of four URGs in HepG2 and Huh7 cells, respectively. *P < 0.05, **P < 0.01, ***P < 0.001.

Sorafenib is the first-line drug for the treatment of advanced liver cancer. We then analyzed the relationship between sorafenib sensitivity and expression of 4 URGs. The IC50 of sorafenib was significantly negatively correlated with the expression of 4 URGs (**Figure 9A**). The HCC patients with higher expression of 4 URGs had lower IC50 values, indicating that patients with high expression of 4 URGs were more sensitive to sorafenib treatment (**Figure 9B**).

To further confirm whether sorafenib could alter the expression level of four URGs, HepG2 and Huh7 cells were treated with different concentrations of sorafenib for 24 h. Consistent with previous results, cell viability was decreased in a dose-dependent manner (**Figures 9C, D**). Additionally, the expression of GOSR2, PDIA6 and SRPRB was gradually decreased, but the expression ATF4 was significantly increased in HepG2 and Huh7 cells following sorafenib treatment (**Figures 9E, F**).

## Discussion

Liver cancer is still a serious public health problem that globally endangers people’s health. HCC is the most common pathological type. At present, the survival rate of HCC patients is still not optimistic, and there is a lack of appropriate clinical prognostic indicators in HCC treatment. It is critical to further understand the pathological molecular mechanisms of HCC and identify appropriate clinical prognostic indicators. UPR plays an important role in maintaining protein homeostasis in cells under numerous intracellular and extracellular stresses and closely associates with oncogenesis (13, 25). In the present study, we construct a prognostic model based on URGs, and this URG-related signature exhibits excellent capability in predicting the prognosis of HCC patients.

We analyzed the role of UPR in the HCC from the expression level and immune cell infiltration, constructed a risk score model, and evaluated the potential value of URGs as clinical prognostic indicators of HCC. Thirty-one URGs were selected for further analyses (**Figure 1**). We analyzed the correlations of 31 URGs in HCC samples and noticed that most UPR genes were positively correlated in both TCGA and ICGC databases. These results were consistent with previous studies. For example, ATF4 expression is positively correlated with CCL2 in both TCGA and ICGC databases (**Figures 1E, F**). Previous studies have shown that transcription factor ATF4 can promote the expression of CCL2, which in turn promotes the infiltration of macrophages in endometrial cancer (26). HSPA5 serves as an important mediator of ER stress response. Both ATF4 and ATF6 are positively correlated with HSPA5 (**Figures 1E, F**). In fact, HSPA5 is an important target gene of ATF4 and ATF6 (27, 28). However, due to significant differences in HCC samples from the two independent databases in terms of race, gender, age and pathological stage, there were also some differences in the correlation results (**Figures 1E, F**). Together, these results imply that UPR genes may interact with and/or positively regulate expression of each other through some unknown molecular mechanisms.

Over the last decade, emerging evidence has revealed that UPR is closely related to tumor initiation, progression, metastasis and chemoresistance. Previous studies have demonstrated that various UPR-related genes, including XBP1s, eIF-2α, CHOP, ATF4 and ATF6, are overexpressed or activated in many tumor cells (29–32). High expression of UPR-related components indicates the deterioration of cancer. UPR also participates in tumor angiogenesis, which brings nutrients and creates favorable living conditions for tumors. XBP1 and ATF4 bind to the VEGF promoter to upregulate its expression, thereby promoting angiogenesis (33). ATF4 also promotes the expression of IL-8 to facilitate tumor angiogenesis (34). Consistent with previous studies, the expression of most URGs was significantly higher in HCC samples than normal samples (**Figure 1**). High expression of UPR genes in patients predicted poor prognosis (35). These results indicate that UPR has potential as clinical prognostic factor in HCC.

UPR not only affects the survival of cancer cells but also participates in tumor immunosuppression. Immunotherapy has become a major therapy for patients with advanced HCC. Cancer cells also recruit immune cells and cause immune cells to lose their original functions, such as destroying the antigen transmission function of dendritic cells (36). Various metabolic stimuli, such as hypoxia, lead to an imbalance in protein homeostasis in immune cells, induce UPR, and interfere with the normal function of immune cells (36). As an important signaling pathway of UPR, the IRE1α-XBP1 axis is involved in tumor immunosuppression and closely related to tumor progression. In the mouse model of ovarian cancer lacking XBP1, ovarian cancer progressed more slowly (37). XBP1 also promotes the accumulation of oxidized fatty acids in cells (38). Some studies have shown that excessive accumulation of oxidized fatty acids inhibits the antigen presentation function of dendritic cells (39). According to our results, there are differences in the characteristics of immune cell infiltration between HCC patients with high expression and low expression of URGs. Interestingly, we observed that the infiltrated abundance of major types of immune cells was increased in C1 compared with C2 (**Figure 4B**). Furthermore, we also uncovered the expression of 50 common immune checkpoint genes, including PD-1, PD-L1 and CTLA4, in different subgroups. We found that the expression of most immunosuppressive factors in C1 was higher than that in C2. Consistently, the TIDE score showed that patients in C1 were more prone to immune escape during immunotherapy (**Figure 4E**). Therefore, UPR may influence the immune cell infiltration and immunotherapy effect in HCC (40). Fortunately, HCC patients in C1 seemed to be more sensitive to drugs than patients in C2 (**Figure 6**).

A recent study has reported that high level of UPR score is related with worse prognosis in HCC patients (41). In the present study, two independent subgroups were clustered using the consensus clustering analysis based on the expression of 31 URGs (**Figure 2B**). There were significant differences in four survival indexes (OS, PFS, DSS and DFS) between C1 and C2 (**Figures 2E-H**). However, we noticed that not all 31 UPR-related genes are significantly related to the prognosis of HCC patients (**Figures 7A, B**), and due to the large number of genes, it is difficult to apply them in clinical practice. To solve this problem, we identified 4 prognostic core genes (SRPRB, PDIA6, GOSR2 and ATF4) from 31 URGs through Cox regression and LASSO analyses. Based on the expression levels of these 4 genes, we calculated the risk score and divided patients into high-risk and low-risk groups. The OS of high-risk group was significantly lower than low-risk group (**Figure 7G**). The AUC values of this risk score model at 1, 3, and 5 years were ≥0.65, suggesting that the integrated prognostic model exhibited a promising predictive ability for the prognosis of HCC patients (**Figure 7H**). The upregulated expression of SRPRB, PDIA6, GOSR2 and ATF4 in HCC tissues was observed not only in the TCGA database but also in the ICGC, GSE36376 and GSE14520 datasets (**Figure S10**). Single-cell RNA sequencing results demonstrated that these genes were expressed in both tumor cells and immune cells (**Figure 8A**). Together, our findings demonstrated the potential clinical significance of the 4-URGs-based classifier as a novel biomarker for outcome prediction and therapy decisions in HCC patients.

To improve the prognosis of HCC patients, a growing number of therapeutic strategies and chemotherapy regimens are gradually emerging. However, systemic chemotherapy for HCC has limited value in clinical practice at present, because only a small portion of patients obtain significant effects according to clinical trial results (42–47). Here, we selected ten representative chemotherapeutic agents and observed that patients in C1 were more sensitive to these drugs. Although HCC samples were clustered into two subgroups with different prognosis, signaling pathways, infiltrated immune cell levels and chemotherapy responses, it is unclear whether chemotherapy affects the prognosis of C1 patients and C2 patients. Therefore, it is necessary to collect a large number of clinical samples to analyze the application potential of classification and analyze the impact of chemotherapy drugs such as sorafenib on the prognosis of patients based on this classification. Sorafenib, as a first-line drug for HCC, has shown strong efficacy in the clinical treatment of liver cancer. In the drug sensitivity analyses, we found that the expression of all core 4 URGs was significantly and negatively correlated with the sorafenib sensitivity in HCC samples, indicating that patients with high levels of 4 URGs may be more sensitive to sorafenib (**Figures 9A, B**). However, when HepG2 and Huh7 cells were treated with sorafenib, SRPRB, PDIA6 and GOSR2 were dose-dependently decreased, and ATF4 was gradually increased after treatment (**Figures 9E, F**). ATF4 is a core stress-induced transcription factor that regulates cellular response to various stresses, such as ER stress, hypoxic condition and nutrient deprivation (48). Accumulating evidence suggested that ATF4 could interact with other transcription factors and activate a wide range of adaptive genes associated with amino acid synthesis, angiogenesis, metastasis and drug resistance (49, 50). ATF4 expression is upregulated in different types of human cancers and correlated with tumor progression and therapy resistance (51). The function of ATF4 in HCC has been preliminarily clarified. Previous studies have shown that ATF4 enhances the level of glutathione in HCC cells and enhances drug resistance (52). The transcription factors YAP/TAZ drive sorafenib resistance in HCC by regulating ferroptosis. Mechanistically, YAP/TAZ promoted the nuclear localization, protein stability and transcriptional activity of ATF4 to induce the expression of SLC7A11 and modulate ferroptotic cell death (51). ATF4 and PERK synergistically promoted the expression of ZNFX1 antisense RNA 1 (ZFAS1) to enhance sorafenib resistance in HCC (53). Therefore, the sorafenib-induced upregulation of ATF4 expression may be related to the generation of drug resistance. ER oxidoreductase PDIA6 is overexpressed in several cancers, including oral squamous cell carcinoma, pancreatic cancer, breast cancer, non-small cell lung cancer (NSCLC) and gastric cancer (GC), and predicts poor outcomes in these cancers (54–57). Knockdown of PDIA6 significantly suppressed cell growth, proliferation, migration and invasiveness and enhanced apoptosis (56, 57). PDIA6 also promoted the deubiquitination of β-catenin and PD-L1 to drive the progression and immune evasion of pancreatic cancer (58). In addition, PDIA6 regulated autophagy and apoptosis in NSCLC cells through the MAP4K1/JNK pathway (55). More importantly, elevated PDIA6 expression is associated with cisplatin resistance in GC and NSCLC cells. Therefore, PDIA6 is a promising target for drug resistance (55). SRBPR has been reported as a prognostic indicator of HCC (59). GOSR2 is reported to be involved in the pathogenesis of progressive myoclonus epilepsy (60). However, the functions of SRPRB and GOSR2 in the development of HCC are still needed to be explored. Based on our RT-PCR results, we speculate that sorafenib may exhibit its anticancer effects partially through downregulating the expression of SRPRB, PDIA6 and GOSR2. However, with the treatment of sorafenib, the decrease in the expression of these genes and increased in the expression of ATF4 gradually reduce the sensitivity of patients to sorafenib, which may also be an important cause of generation of sorafenib resistance.

In summary, we identified two clusters according to UPR-related gene expression, and constructed and verified a novel UPR-related signature with excellent prognostic potential using different independent datasets. Additionally, this signature was significantly associated with the tumor immune microenvironment and immunotherapeutic/chemotherapeutic responses in HCC patients. In summary, this UPR-related signature serves as a clinical prognostic indicator of HCC, clarifies the molecular mechanism of prognosis, and provides guidance for the clinical management of HCC patients.

## Data availability statement

The original contributions presented in the study are included in the article/**Supplementary Material**. Further inquiries can be directed to the corresponding authors.

## Author contributions

Study concept and design: KT and HG. Acquisition of data: KT, HG, SZ, BZ and YS. Analysis and interpretation of data: KT, HG, SZ, BZ, YS, XL and MW. RT-PCR analysis: HG, MW and SZ. Statistical analysis: KT, HG, SZ and HW. Drafting of the article: KT. Critical revision and final approval of the article: HG, YF and KT. Obtained funding: KT. Study supervision: KT. All authors contributed to the article and approved the submitted version.

## Supplementary material

Supplementary Figure 1 Flow chart of this study.

Supplementary Figure 2 Identification of DEGs between HCC samples and normal samples in the TCGA database. **(A)** Heatmap showing the identified DEGs between HCC samples and normal samples. **(B)** 31 URGs were differentially expressed in HCC samples and normal samples in the TCGA database. ****P* < 0.001.

Supplementary Figure 3 31 URGs were differentially expressed in HCC and normal samples in the ICGC database. **(A)** The expression of 31 URGs between HCC samples and normal samples was analyzed according to the ICGC database. **(B)** Heatmap showing the expression of 31 URGs in HCC samples and normal samples. ****P* < 0.001.

Supplementary Figure 4 Signaling pathway enrichment analysis was performed using the Metascape tool.

Supplementary Figure 5 The expression of ferroptosis- and m^6^A-related genes in C1 and C2. **(A)** The expression of ferroptosis-related genes in C1 and C2. **(B)** The expression of m^6^A-related genes in C1 and C2. **P* < 0.05, ***P* < 0.01, ****P* < 0.001.

Supplementary Figure 6 Infiltrated abundance of immune cells based on the XCell algorithm in C1 and C2. **(A, B)** Comparison of the infiltrated abundance of each immune cell in C1 and C2. **(C)** The proportion of immune cells in each HCC sample. **P* < 0.05, ***P* < 0.01, ****P* < 0.001.

Supplementary Figure 7 Cox regression analysis of 4-URG prognostic signature to validate the risk score model. **(A)** The risk score model was established by Cox regression analysis according to the ICGC database. **(B)** KM analysis of OS in high-risk group and low-risk group. **(C)** AUC of time-dependent ROC curves was examined to test the reliability of the risk score model according to the ICGC database.

Supplementary Figure 8 Univariate and multivariate Cox regression analysis and nomogram construction. **(A)** Forest plot showing the result of univariate Cox regression analysis. **(B)** Forest plot showing the result of multivariate Cox regression analysis. **(C)** A nomogram integrating prognostic characteristics was used to predict 1-, 3-, and 5-year OS in HCC. **(D)** Calibration curves were used to examine the agreement between the actual and predicted 1-, 3-, and 5-year OS.

Supplementary Figure 9 KM analysis was performed to estimate the OS in high-risk group and low-risk group with different clinicopathological parameters.

Supplementary Figure 10 The expression of four URGs in different datasets. **(A, B)** The expression of the 4-URG signature in HCC patients based on gene-chip data and RNA-seq data in the TNMplot database. **(C)** Expression of four URGs in HCC samples and normal samples in the GSE36376 dataset. **(B)** Expression of four URGs in HCC samples and normal samples in the GSE14520 dataset. ****P* < 0.001.
