# Supplementary figures and images for: Immunogenic landscape and risk score prediction based on unfolded protein response (UPR)-related molecular subtypes in hepatocellular carcinoma
Source: Front Immunol. 2023 Jun 30;14:1202324. doi: 10.3389/fimmu.2023.1202324 (PMC10348016; doi:10.3389/fimmu.2023.1202324)

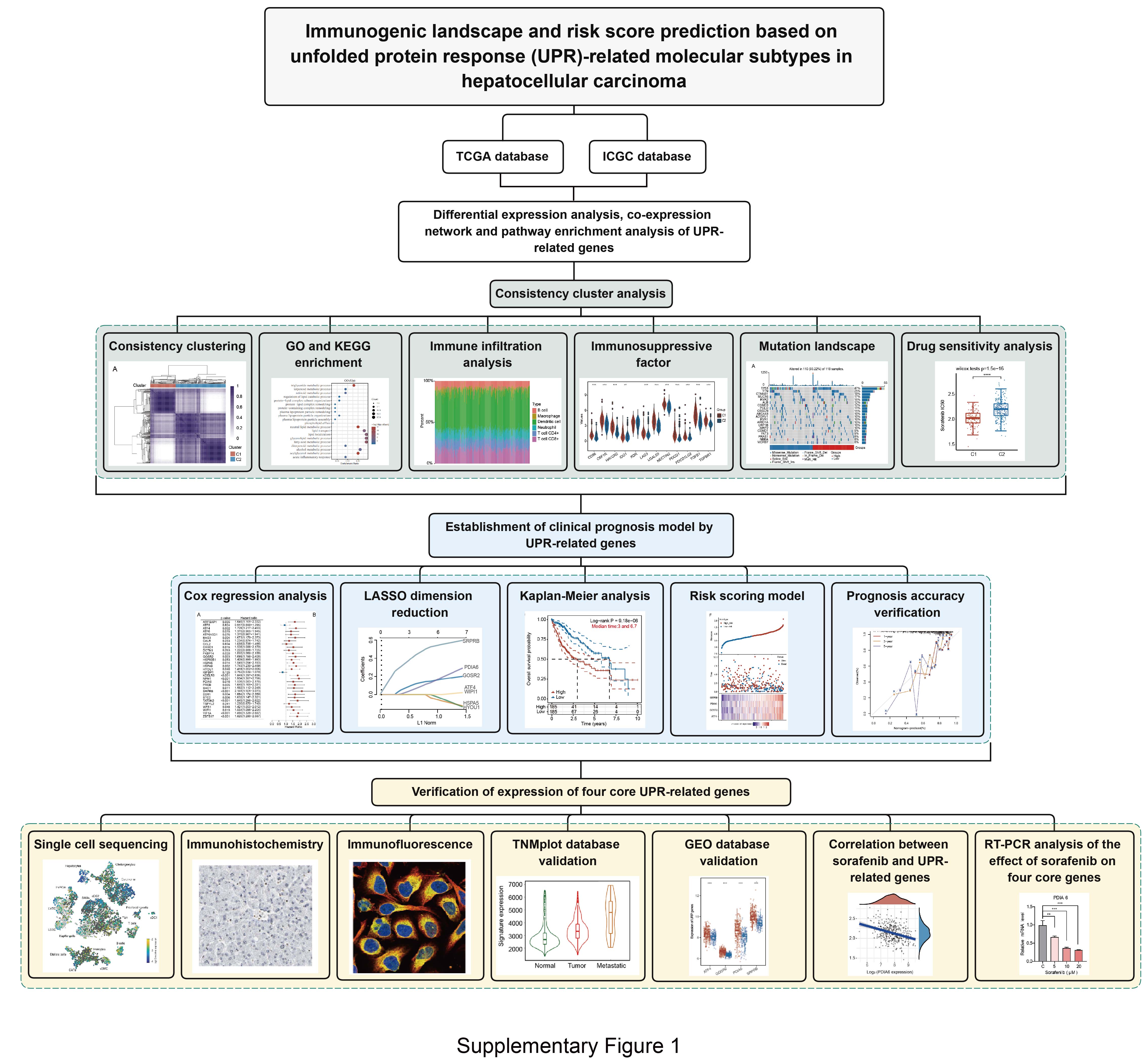

Supplement: Supplementary Figure 1 — Flow chart of this study. [file Image_1.jpeg]

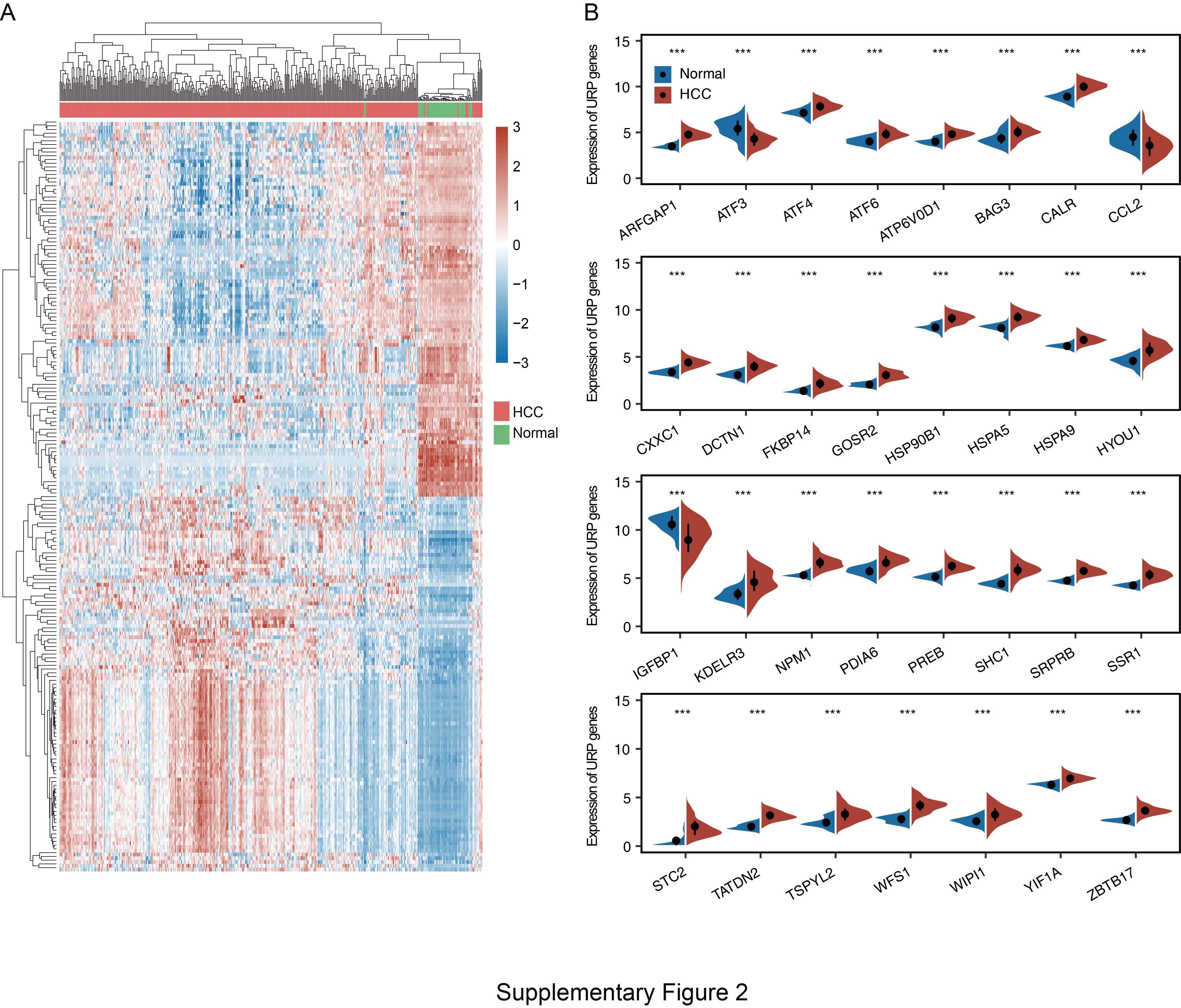

Supplement: Supplementary Figure 2 — Identification of DEGs between HCC samples and normal samples in the TCGA database. (A) Heatmap showing the identified DEGs between HCC samples and normal samples. (B) 31 URGs were differentially expressed in HCC samples and normal samples in the TCGA database. ***P < 0.001. [file Image_2.jpeg]

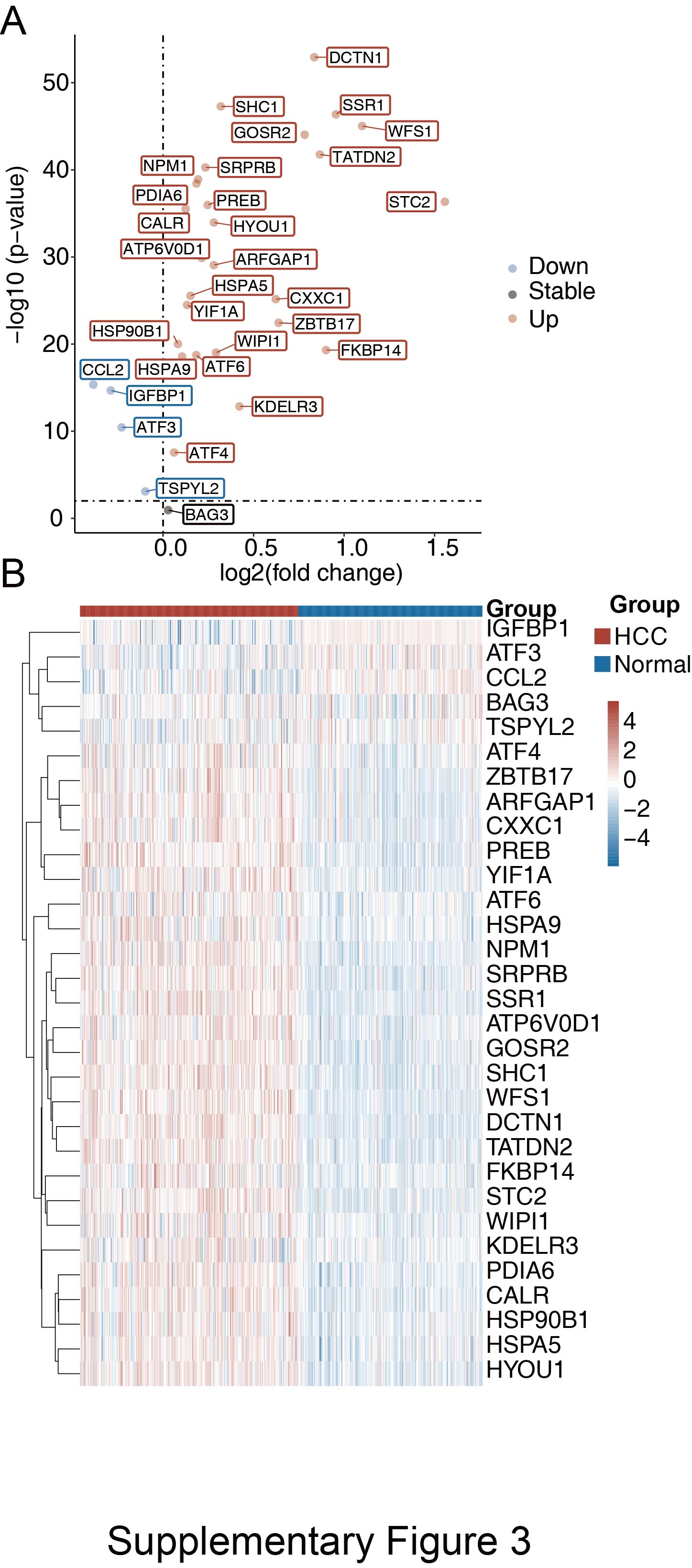

Supplement: Supplementary Figure 3 — 31 URGs were differentially expressed in HCC and normal samples in the ICGC database. (A) The expression of 31 URGs between HCC samples and normal samples was analyzed according to the ICGC database. (B) Heatmap showing the expression of 31 URGs in HCC samples and normal samples. ***P < 0.001. [file Image_3.jpeg]

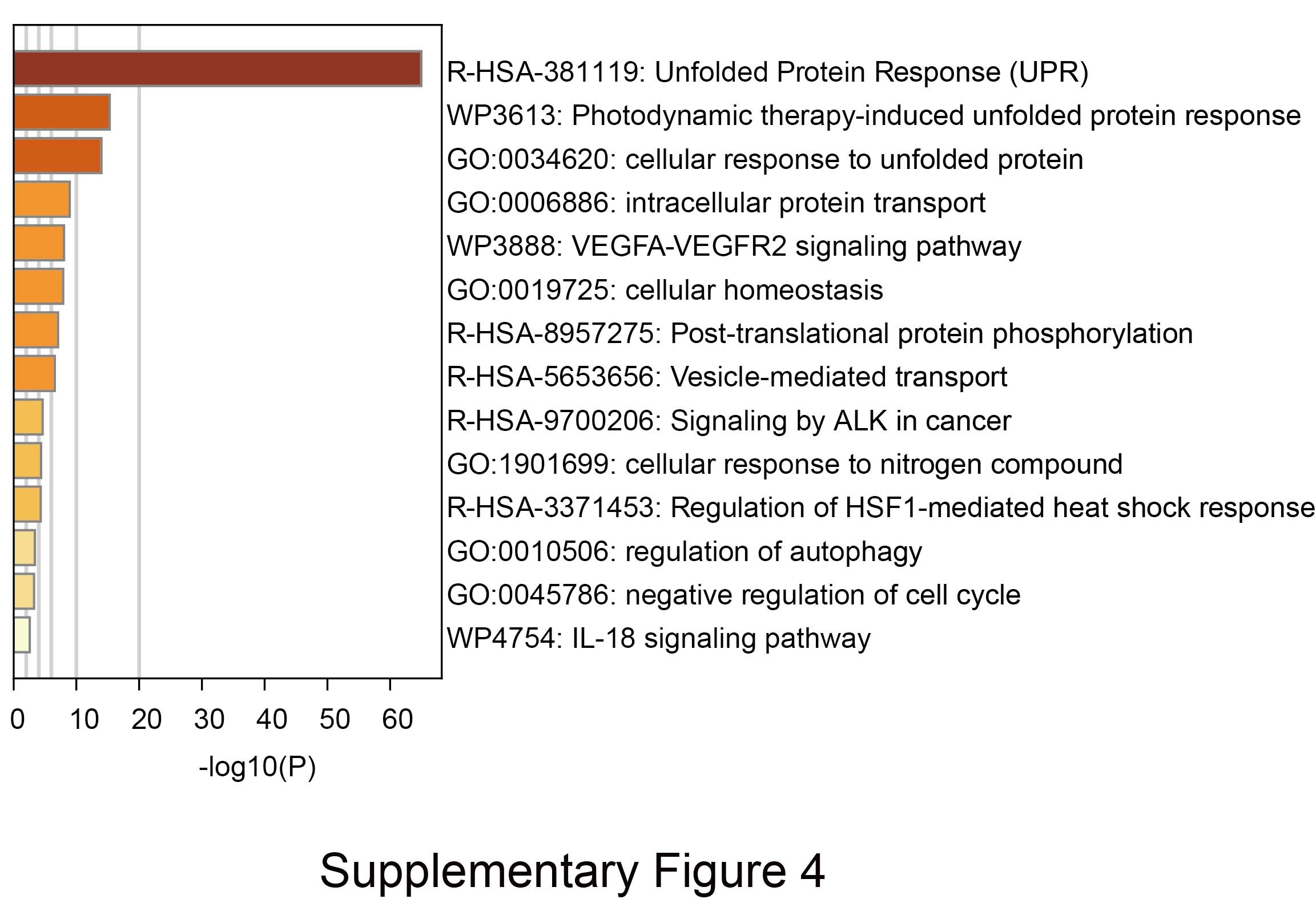

Supplement: Supplementary Figure 4 — Signaling pathway enrichment analysis was performed using the Metascape tool. [file Image_4.jpeg]

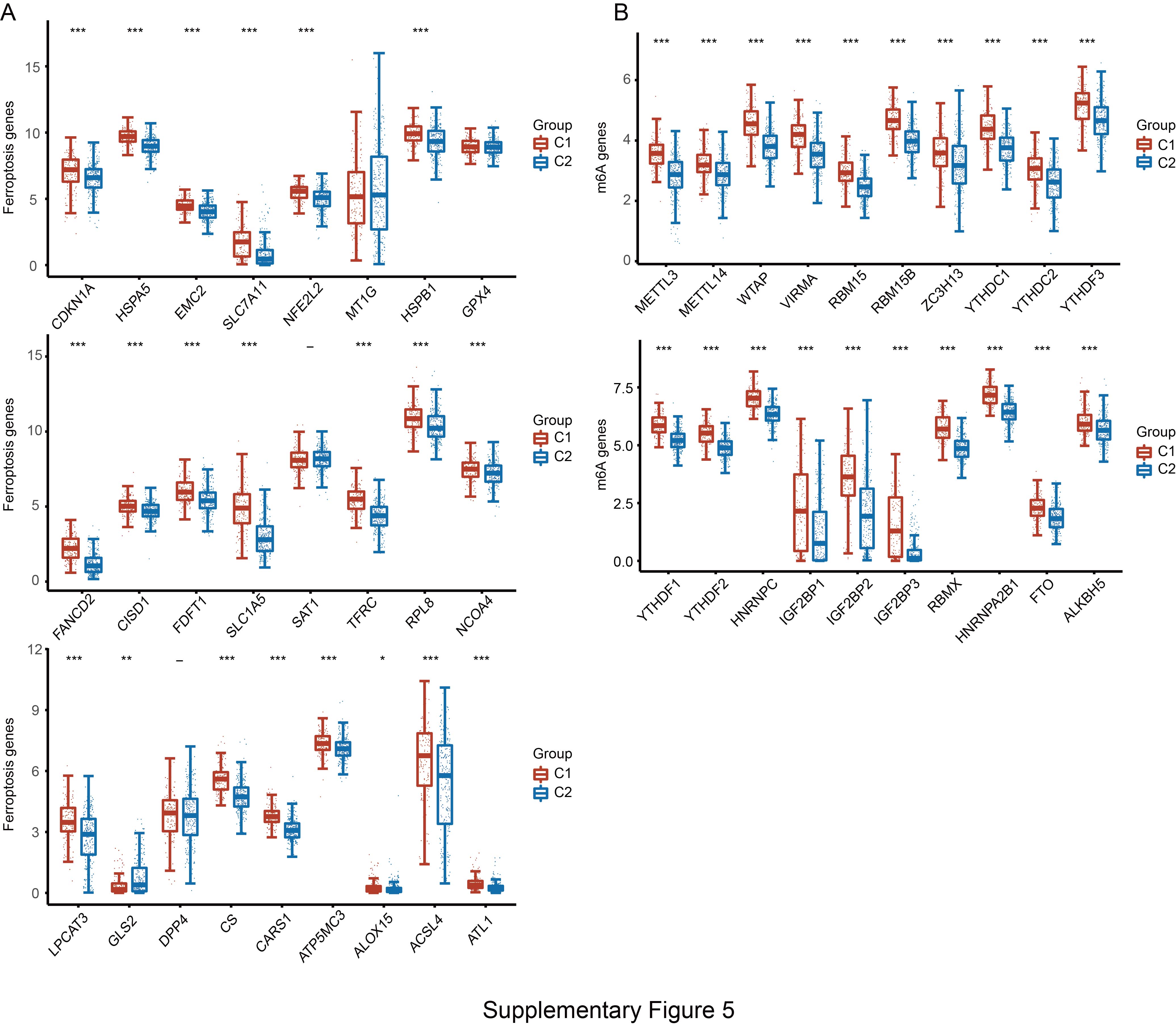

Supplement: Supplementary Figure 5 — The expression of ferroptosis- and m6A-related genes in C1 and C2. (A) The expression of ferroptosis-related genes in C1 and C2. (B) The expression of m6A-related genes in C1 and C2. *P < 0.05, **P < 0.01, ***P < 0.001. [file Image_5.jpeg]

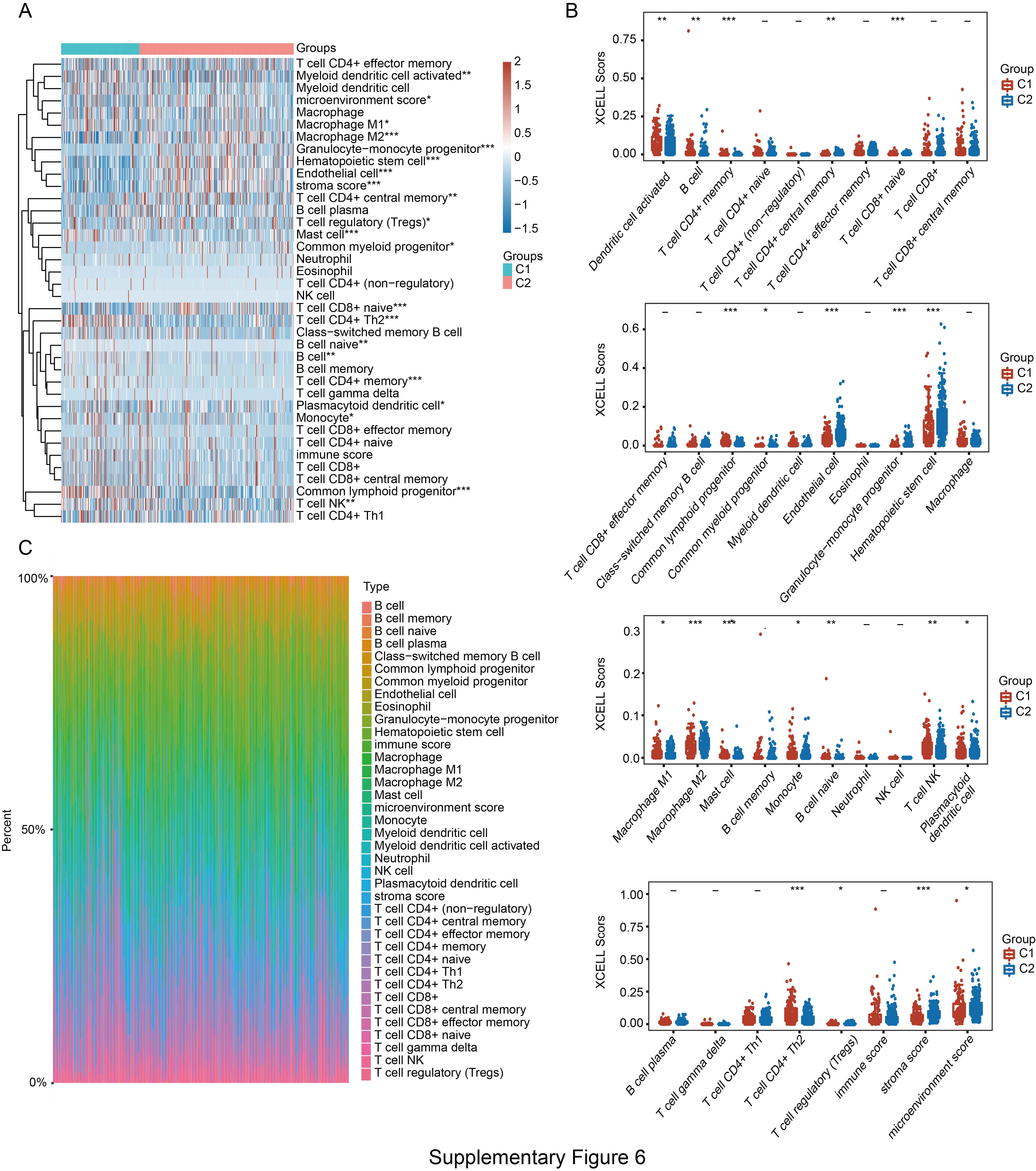

Supplement: Supplementary Figure 6 — Infiltrated abundance of immune cells based on the XCell algorithm in C1 and C2. (A, B) Comparison of the infiltrated abundance of each immune cell in C1 and C2. (C) The proportion of immune cells in each HCC sample. *P < 0.05, **P < 0.01, ***P < 0.001. [file Image_6.jpeg]

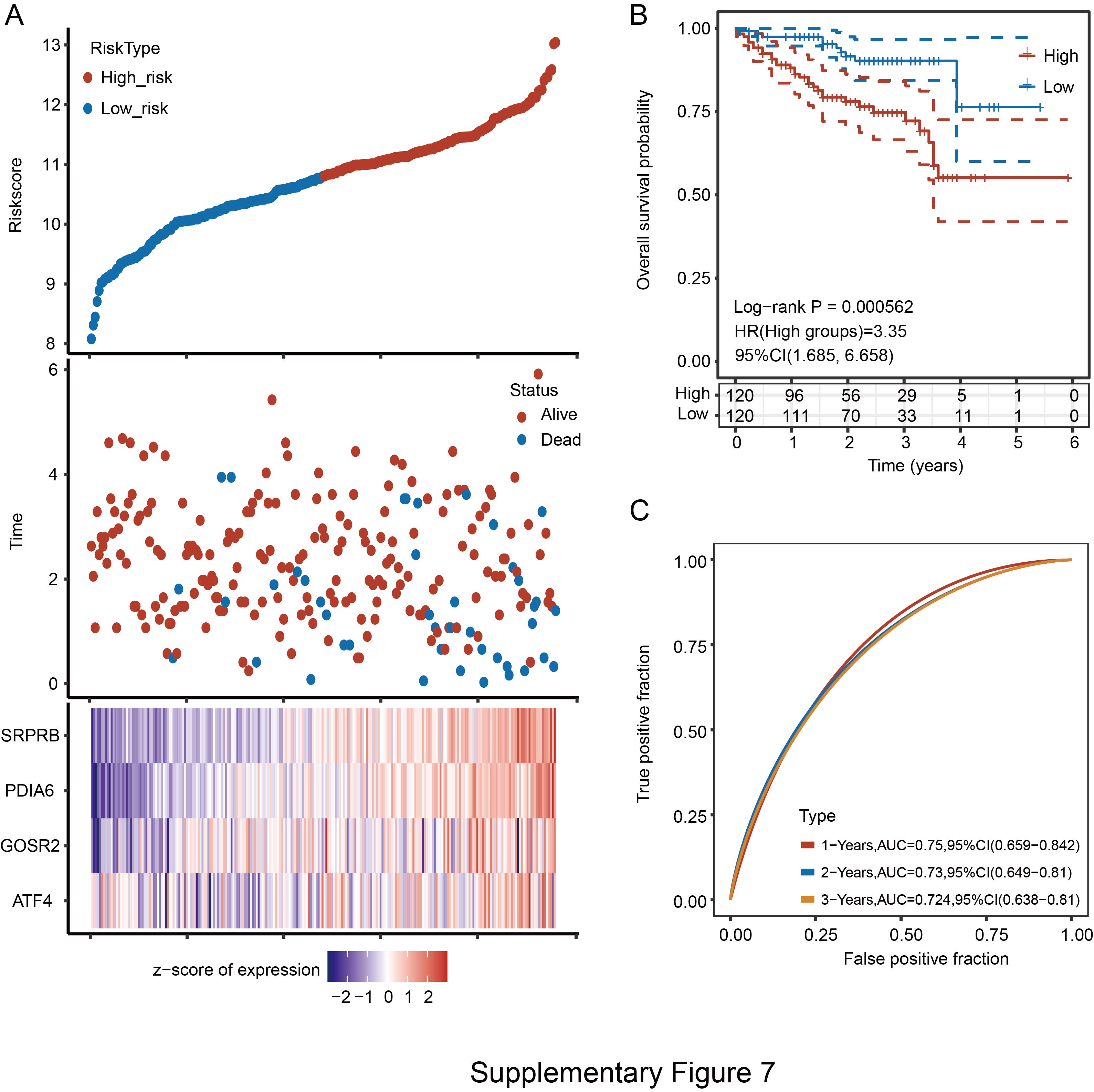

Supplement: Supplementary Figure 7 — Cox regression analysis of 4-URG prognostic signature to validate the risk score model. (A) The risk score model was established by Cox regression analysis according to the ICGC database. (B) KM analysis of OS in high-risk group and low-risk group. (C) AUC of time-dependent ROC curves was examined to test the reliability of the risk score model according to the ICGC database. [file Image_7.jpeg]

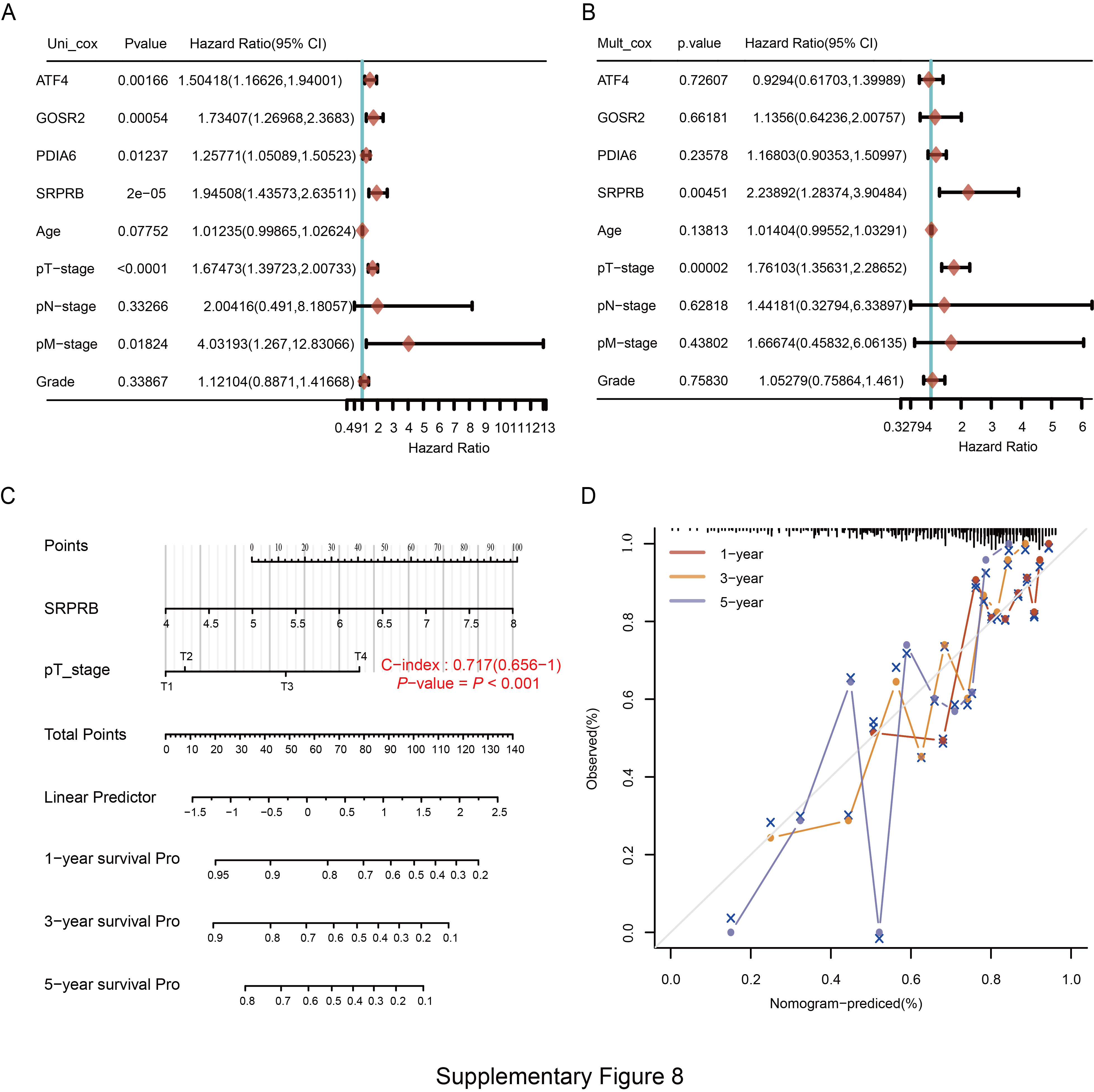

Supplement: Supplementary Figure 8 — Univariate and multivariate Cox regression analysis and nomogram construction. (A) Forest plot showing the result of univariate Cox regression analysis. (B) Forest plot showing the result of multivariate Cox regression analysis. (C) A nomogram integrating prognostic characteristics was used to predict 1-, 3-, and 5-year OS in HCC. (D) Calibration curves were used to examine the agreement between the actual and predicted 1-, 3-, and 5-year OS. [file Image_8.jpg]

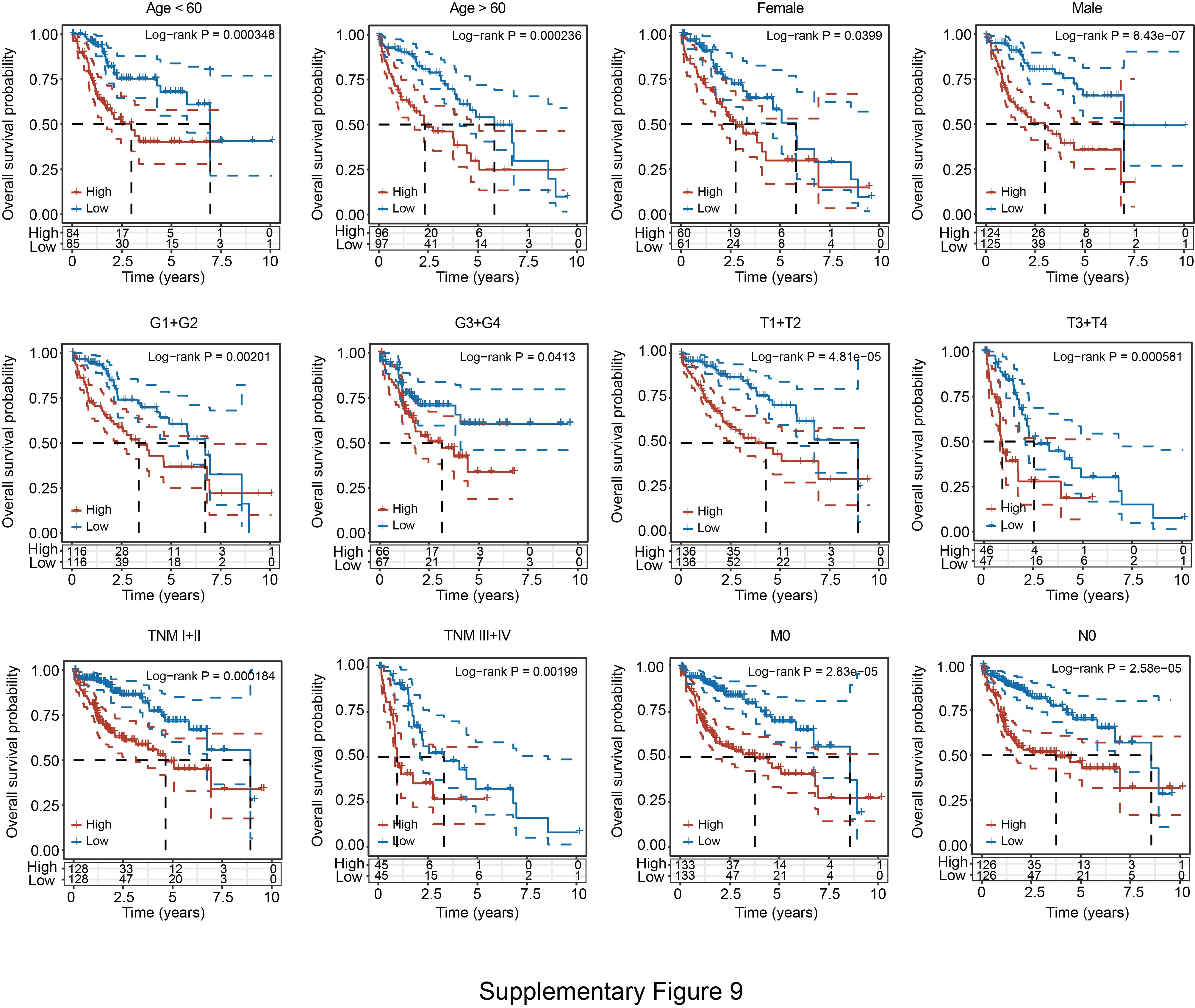

Supplement: Supplementary Figure 9 — KM analysis was performed to estimate the OS in high-risk group and low-risk group with different clinicopathological parameters. [file Image_9.jpeg]

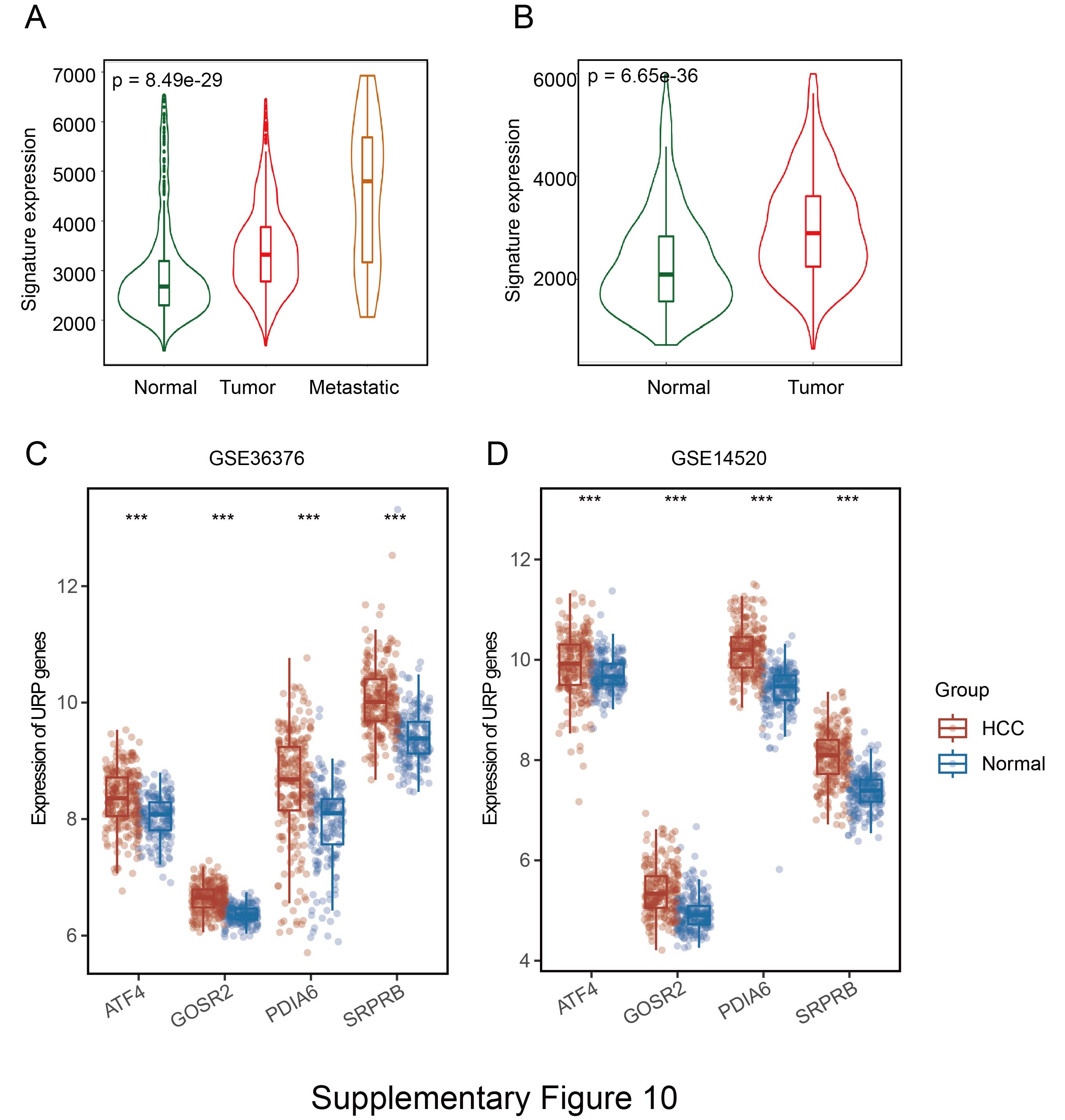

Supplement: Supplementary Figure 10 — The expression of four URGs in different datasets. (A, B) The expression of the 4-URG signature in HCC patients based on gene-chip data and RNA-seq data in the TNMplot database. (C) Expression of four URGs in HCC samples and normal samples in the GSE36376 dataset. (B) Expression of four URGs in HCC samples and normal samples in the GSE14520 dataset. ***P < 0.001. [file Image_10.jpeg]
